# Interesting Analyses of Articles in Foreign Journals

**Published:** 1864-10

**Authors:** 


### INTERESTING ANALYSES OF ARTICLES IN FOREIGN JOURNALS.

*Addison's Disease.*—Two specimens of bronzing of the skin and disease of the supra-renal capsules have recently been brought to the notice of the Pathological Society of London: A young woman aged twenty-three years, was admitted with symptoms of chorea. A month before admission into the hospital she suddenly fell down and could not rise without assistance. She had no convulsions paralysis, or loss of consciousness. This was followed by low fever for two weeks. She then stated that she could not use her left hand, nor afterwards her left foot, as before. Choreic movements came on and continued until her admission, at which time they were observed chiefly in the leg, hand and face. She became gradually weaker, and in three days was delirious, getting out of her bed and falling down several times. A certain bronzing of the skin was at first noticed, *which* was attributed to exposure to the sun, although it was afterwards ascertained that she had not been exposed to the solar rays, as she was of a sedentary occupation, and much confined to the house. She gradually sank, and died nine days after admission. At the autopsy, the supra-renal capsules were found to be extensively diseased; in neither was there any traces of their original healthy structure, but the microscope showed them to consist of scrofulous material. The weight of one capsule was five drachms and two grains, whilst that of the other was two drachms and six grains. On examining the spinal cord, a small tumor was discovered upon its posterior aspect in the lumbar region, springing from the interior of the cord itself. There was an enlargement of the latter higher up, but no farther tumor. The melasma was more marked over the face and arms than elsewhere, and this had been noticed by the patient's friends before death. The brain and other organs were healthy. The case may be looked upon as a true example of Addison's disease, with the accidental complication, if we may so call it, of tumor and enlargement of the spinal marrow, which it is fair to assume gave rise to the nervous symptoms resembling chorea. Excluding the spinal disease, no other change was noticed after death to explain the melasma but the diseased condition of the supra-renal capsules. Differing from the preceding case, the melasma in the following example was in some parts of the body so extreme as to simulate the color of the skin of a moderately dark negro. The specimen of it selected for illustration before the Society seemed a very dark brown, approaching to black. Beyond some slight deposit of tubercle in the apices of both lungs, no parts were found affected but the supra-renal capsules, the case thus constituting a true example of Addison's disease. The patient was a boy of fourteen years, in whom the melasma appeared four months before his entrance into the hospital, previous to which time he had enjoyed excellent health. Pain in the right hypochondriac and umbilical regions was a prominent feature. The parts chiefly affected with melasma were the abdomen and loins, and inner side of both thighs. Generous diet, steel, and other means were unavailingly employed, and a fatal result ensued after a stay in the hospital of about five days. At the autopsy, with the exception of the tubercle at the apices of both lungs, the supra-renal capsules were the only parts found diseased. They were transformed into tuberculous matter, mixed with calcareous particles, and of their normal dimensions. What is remarkable, is the disease running its course, in a mere lad, in the very short space of four months and a half, previous to which the patient had been in very good health. Usually, Addison's disease extends over a much longer period of time.

*Sir Benjamin Brodie on Homœopathy.*—* * * To a certain extent homœpathy can be productive of no great harm; and indeed, considering it to be no treatment at all, whenever it is a substitute for bad treatment, it must be the better of the two. But there is great harm, nevertheless. There are numerous cases in which spontaneous recovery is out of the question; in which sometimes the life or death of the patient, and at other times the comfort or discomfort of his existence for a long time to come, depends on the prompt application of active and judicious treatment. In such cases homœpathy is neither more nor less than a mischievous absurdity; and I do not hesitate to say that a very large number of persons have fallen victims to the faith which they reposed in it, and to the consequent delay in having recourse to judicious remedies. It is true that it very rarely happens, when any symptoms show themselves which give real alarm to the patient or his friends, that they do not dismiss the homœpathist and send for a regular practitioner; but it may well be that by this time the mischief is done, the case being advanced beyond the reach of art.

It cannot be otherwise than provoking to those who have spent years of the best part of their lives in endeavoring to make themselves well acquainted with disease in the wards of an hospital, to find that there are some who resort to them only when their complaints have assumed a more painful or dangerous character; while they are set aside in ordinary cases, which involve a smaller amount of anxiety and responsibility, in favor of some homœpathic doctor, who probably never studied disease at all. But it cannot be helped. In all times there have been pretenders, who have persuaded a certain part of the public that they have some peculiar knowledge of a royal road to cure, which those of the regular craft have not. It is homœopathy now; it was something else formerly; and if homœopathy were to be extinguished, there would be something else in its place. The medical profession must be contented to let the thing take its course; and they will best consult their own dignity and the good of the public by saying as little as possible about it. * * * After all, the harm done to the regular profession is not so great as many suppose it to be; a very large proportion of the complaints about which homœpathists are consulted being really no complaints at all, for which a respectable practitioner would scarcely think it right to prescribe.

*Diabetes.* By W. G. CARTER, M. R. C. P.—I am inclined to the physiological theory inaugurated by M. Bernard, and to ascribe the primary cause of the disease to a deficiency of nervous power from some morbid impression at or about the origin of the pneumogastric nerves. For what are the most common complications and secondary disorders of this complaint? Tubercular phthisis, dyspepsia from increased irritability of the stomach, anasarca, and apoplexy; to which may be added, according to M. Bernard, the increased glucogenic function of the liver by an injury to the medulla in the region of the fourth ventricle. And that it is a disease of the nervous centres, rather than of any secernent or excernant organ, the analogy of it to others which we know to be so, as well as the nature of the malady itself, afford striking if not positive evidence of the correctness of my assumption. The celebrated Dr. Wollaston refered the production and distinction of all the secretions to that change in the quality or intensity of the nervous power transmitted to it; and Dr. Van der Kolh refers all the spasmodic or convulsive movements to the ganglionic cells of the medulla oblongata, which as reflex ganglia, possess the peculiar property, that, when once brought into an excited condition, they may more or less suddenly discharge themselves and communicate their influence to the different nervous filaments just as we see to be the case with electric batteries or in the phenomenon of an electric fish. Moreover, the treatment admitted on all sides to have been most efficacious, offers to my mind a still farther negative evidence of the disease being nervous rather than either humoral or chemical in its character. Dr. Latham frequently prescribed phosphorus with success, and we know what a powerful aphrodisiac that is when there is a deficiency of nervous power. Cullen was so satisfied with this doctrine that we find diabetes ranged in his class neuroses, and ordo spasmi, immediately before hysteria and hydrophobia. Acting under these impressions, I prescribed as a nervous tonic, a pill containing the following ingredients: Sulphate of iron, extract of nux vomica, compound extract of colocynth, and hyoscyamus—to be taken night and morning; with a mixture of phosphate of soda and infusion of gentian, twice a day; avoiding as much as possible all vegetables, and recommending a diet of a highly azotised nature. Under this treatment the urine lost its high specific gravity; the thirst became much less; and in a few weeks not the slightest reaction could be obtained by the solution of Barresevil, or any other process with which I am acquainted, and the patient recovered his usual health and spirits

*Tetanus of Fourteen Months' Duration.*—Mr L—— aged thirty-six, a strong fisherman, but with hæmorrhagic tendency, early in December, had a cough of unusual violence. An act of coughing suddenly ceased, and the head was at the same time spasmodically thrown backwards, with great pain in the neck. Complete tetanus gradually developed itself in every form and lasted, with such intermissions as were evidently the effect of remedies, till the middle of January of the second year afterwards. Treatment of every kind was tried. The prolongation of life seemed to be due to the free breathing of chloroform and the constant use of morphia both by the mouth and by injection under the skin. A small clot was found on the front of the cervical part of the spinal cord.—*Lancet.*

*Tobacco.*—Recently at a meeting of the Society of Emulation at Rouen, a paper was read by Dr. Dumesnil on Tobacco and the effects attending its use, of which the following is an abstract: "The custom of smoking is spreading through the whole world The tobacco-producing countries having the greatest difficulty in providing for local wants. In America, the consumption augments more rapidly than the supply. According to late statistics, the quantity of tobacco used in the New World annually, is in weight equal to the bread consumed by ten millions of individuals in England. England, a country which does not consume tobacco, yearly consumes 36,000 000 lbs. of that plant drawn from America; and during the last ten years her consumption has increased one-fourth. In Hamburg, the population of which is only 150 000, as many as 40 000 cigars are consumed per day. In Denmark, the average annual consumption is 4 lbs. per head for the whole population. In Holland the proportion is still higher. In Austria, the cultivation of the tobacco plant occupies 100,000 acres of good land. In 1854, the consumption of tobacco in the whole world amounted to 500,000,000 lbs., being an average of 9 oz. per each person. Calculating that tobacco contains on an average 3 per cent. of nicotine, it will be seen that there are annually consumed on the globe 15,180 000 lbs. of a poison of which a few drops are sufficient to cause death."

*Case of Double Uterus with Simultaneous Gestation.*—Mr Grace was summoned to see a patient in labor for the fourth time, aged twenty-six. Twice previously there had been premature birth; the third child did not live. When first seen by Mr. Grace, labor had been going on for fifteen hours; the waters had escaped. On examination, a hand was found presenting in the vagina, and the os about half dilated; but lying posterior to this, another os was discovered, with the head of a child presenting septum between the two half an inch thick, and extending up as far as could be reached. The anterior os was dilated, the child turned, and delivery effected. The placenta then followed. The child was dead, and apparently seven months old. The posterior os was next dilated, turning effected, and a live child extracted, which survived only a few hours. The placenta of the second child was expelled without difficulty. Both children were females, equal in development. No flooding or other complication interfered with the perfect recovery of the patient.

In the elaborate work of Kussmaul on the Malformations of the Uterus, which contains a large collection of cases of various kinds, there are only two recorded precisely similar to this. Abortion and premature labor seem especially liable to occur in cases of double uterus.

*Nicotine found in the Viscera of a Snuff-Taker.*—M Morin, of Rouen, anxious to ascertain whether nicotine could be detected in the viscera, subjected the lungs and liver of a snuff-taker, who had died at seventy, to a careful analysis, and found the alkaloid just mentioned. Portions of the lungs and liver were reduced to a pulp and soaked in distilled water, slightly acidulated for the lungs with sulphuric acid, and for the liver with oxalic acid "Several days afterwards, the liquor was filtered through paper free from carbonate of lime, then concentrated to a third of its volume and filtered again to free it from the flakes which had formed. It was now once more evaporated and treated with pure alcohol, when fresh flakes formed. The alcohol was then removed by heat after filtration The residue was mixed with a small quantity of pure potash On cooling, sulphuric ether was added; and, after a few hours the decanted liquor was evaporated in vacuo. The substance now obtained having the smell and the acrid taste of nicotine, was treated by the bichloride of mercury, chloride of platinum, tannin, biniodide of potassium, the salts of copper and lead, and with all were the re-actions of nicotine obtained.

